# Non-linear association of cystatin C and all-cause mortality of heart failure: A secondary analysis based on a published database

**DOI:** 10.3389/fcvm.2022.930498

**Published:** 2022-09-06

**Authors:** Tao Zheng, A-Mei Tang, Yuan-Lei Huang, Jin Chen

**Affiliations:** ^1^Department of Cardiology, The First Affiliated Hospital of Guizhou University of Traditional Chinese Medicine, Guiyang, Guizhou, China; ^2^Department of Cardiology, Affiliated Hospital of Guizhou Medical University, Guiyang, Guizhou, China

**Keywords:** CysC, heart failure, all-cause mortality, nonlinear, association

## Abstract

**Background:**

Prior reports have revealed that basal Cystatin-C (CysC) is positively associated with all-cause death in patients with heart failure (HF). Yet, this positive association is not necessarily generalizable to Chinese HF patients due to methodological limitations and lack of data from Chinese patients.

**Materials and methods:**

We performed secondary data mining based on a retrospective cohort dataset published on the internet. This dataset contains 2008 patients with HF who were admitted to a tertiary hospital in Sichuan Province, China from 2016 to 2019. The exposure variable was baseline CysC and the outcome variable was all-cause death on day 28, day 90, and month 6. Covariates were baseline measurements, including demographic data, drug use, comorbidity score, organ function status (heart, kidney), and severity of heart failure.

**Results:**

Among 1966 selected participants, the mortality rates at 28 days, 90 days and 6 months were 1.83% (36/1966), 2.09% (41/1966) and 2.85% (56/1966) respectively. After adjustment for confounders, the non-linear associations between CysC and all-cause deaths were observed. We calculated the inflection points were about 2.5 mg/L of CysC. On the right of inflection point, each increase of 1 mg/L in CysC was associated with an increase in the risk of 28-day mortality (Relative risk [RR], 2.07; 95% confidence interval [CI], 1.09 to 3.93; *P* = 0.0266), 90-day mortality (RR, 2.51; 95% CI, 1.38 to 4.57; *P* = 0.003), and 6-month mortality (RR,2.25; 95% CI, 1.37 to 3.70; *P* < 0.001).

**Conclusion:**

Our findings suggest that values about 2.5 mg/l of cystatin could be a danger threshold for the short-term risk of death in heart failure. Exceeding this threshold, for every 1 mg/L increase in CysC, the risk of all-cause mortality increased by more than one time.

## Introduction

Heart failure (HF) is a serious global health issue that requires urgent attention (1). It has long been regarded as the only cardiovascular disease with a rising global incidence and prevalence (2). The weighted prevalence of HF in male and female inhabitants over 35 years of age in China is 1.3%. The number of HF patients in China is reported to be above 13.70 million (3) due to the country’s vast population. HF not only causes serious physical and psychological impairment to sufferers, but it also places a financial strain on society as a whole (4). According to epidemiological statistics from Western nations, the 5-year death rate of HF is significantly greater than that of several cancers (5). In China, the prevalence of HF has grown sixfold in the last ten years. The mortality rate among individuals with severe HF might reach 50% (6).

Creatinine is routinely used in the early risk stratification of heart failure (7, 8). However, blood creatinine levels are affected by age, muscle mass, gender, and race (9). As a result, cystatin C (CysC) is used in clinical settings. CysC levels are more sensitive than creatinine levels and are less affected by age, gender, or ethnicity (10). Furthermore, past research reveals that CysC has a higher predictive ability than creatinine in predicting cardiovascular-related mortality and other adverse events in the elderly, coronary heart disease (CHD) population, and chronic heart failure (HF) population (11–13). A meta-analysis revealed that greater CysC levels were related to an increased risk of rehospitalization and death in HF patients (14). This data, however, is primarily from Western countries. Data on Chinese patients with HF, on the other hand, is sparse. Furthermore, none of this research looked into the possibility of a non-linear relationship. If an HF prognostic model based on CysC is created in the future, this overlooked non-linear connection is expected to impair model performance (15).

In light of this, researchers undertook this study to look at the link between CysC and the risk of all-cause death in Chinese HF patients. This research will give clinical data as well as fresh insights into the relationship between CysC and outcomes.

## Participants and methods

### Data resource

The data used in this analysis came from an online database (16). The database was generated at Zigong Fourth People’s Hospital. This retrospective cohort comprises a number of 2008 patients with HF who were admitted to Zigong Fourth People’s Hospital between December 2016 and June 2019. The information was obtained from the electronic medical record system of the hospital. A total of 166 variables have been included in the database, including demographics, baseline clinical features, comorbidities, laboratory findings, medications, and clinical outcomes (heart failure readmissions and all-cause death). On the PhysioNet (10.13026/8a9e-w734) website, investigators can retrieve this database for free. Please see “Reference 16” for further information on this database.

### Study population

**FIGURE 1:**
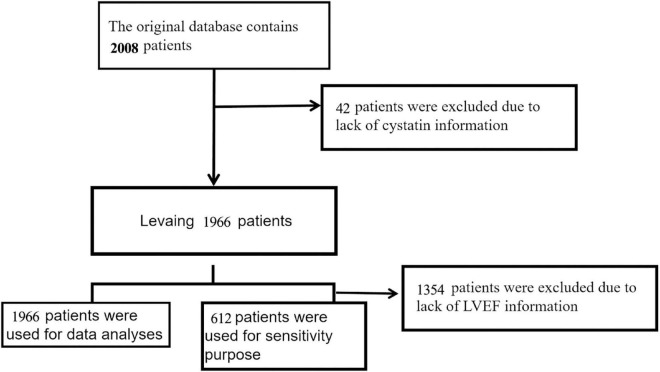
The flowchart of patients selection.

Between December 2016 and June 2019, a total of 2008 adult patients with confirmed HF were included in this retrospective cohort. The European Society of Cardiology (ESC) criteria are used to diagnose heart failure (17). In Figure 1, we explain and demonstrate the patient selection process. 42 patients were ruled out because their CysC was invalid. As a result, the total number of patients in the final dataset was 1966. The database builders have received approval from their hospital’s ethical committee (Approval Number: 2020-010) (16). Patient informed consent was waived because this is a retrospective cohort research with no personal information about the participants (16).

### Variables

#### Outcome variable

The main outcome variables presented in the original database were all-cause mortality at day 28, day 90, and sixth months. Outcome information was not missing. The above outcome variables were recorded as dichotomous variables. Among them, survival is recorded as 0, and non-survival is recorded as 1.

#### Exposure variable

In this study, CysC measured at baseline (admission) was set as an exposure variable and recorded as a continuous variable. Supplementary Figure 1 shows the frequency distribution of Cys-C in the surviving or non-surviving group of HF patients at each time point (histogram).

#### Covariates

The covariates utilized for adjustment in this study were chosen *a priori* based on earlier research that looked into risk factors for HF prognosis. Furthermore, the researchers combined their own clinical experience to arrive at the following characteristics as covariates:

1) Demographic-related indicators: age (≤ 70 years old/ > 70 years old) and sex (female/male);
2) Comorbidities: Charlson comorbidity index score;
3) Drug variables: diuretic use (yes/no), inotropes use (yes/no), statin use (yes/no), vasodilator use (yes/no), Ras-blocker use (yes/no);
4) Heart failure markers: B-type natriuretic peptide, high-sensitivity troponin (hs-CTnT);
5) Other: body mass index (BMI), New York Heart Association classification (NYHA) (II + III/IV), number of hospitalizations (0–1 years/ > 1 years).

It should be noted that: (1) because BNP and hs-CTnT both have a skewed distribution and a skewed residual distribution, the natural logarithm transformation was applied. (2) We created Ras-blockers by combining angiotension converting enzyme inhibitors, angiotensin II receptor blockers, and spironolactone, all of which have similar pharmacological effects.

### Missing data addressing

In this study, the percentage of missing data was less than 5% (Supplementary Table 1). However, when we merged the missing data for each patient, we lost 171 cases (8 percent) from the sample. As a result, to reduce the risk of selection bias caused by missing data, multiple imputation by chained equations, an iterative imputation approach, was adopted (mice package). The five data sets were constructed and sensitivity assessments were performed on the completed data (pre-imputation). Rubins rule was used to generate the final pooled RR values.

### Statistical analysis

We used means and standard deviations to summarize continuous variables, whereas numbers and proportions were used to summarize categorical variables. In addition, we divided HF patients into four groups based on their CysC quartile. Chi-square tests for categorical variables and one-way ANOVA for continuous variables were used to analyze differences between the four groups. The link of CysC with the 28-day, 90-day, and 6-month risks of all-cause mortality was investigated using multivariable logistic regression. model 1 (no covariates were adjusted for), model 2 (only adjusted for age and sex), and model 3 (adjusted for all covariates presented in Table 1) were all displayed at the same time.

**TABLE 1:** Baseline characteristics of patients with heart failure grouped by cystatin (quartile).

	Q1 (0.23–1.20)	Q2 (1.21–1.54)	Q3 (1.55–2.19)	Q4 (2.20–7.06)	*P*-value
N	486	488	498	494	
BMI, mean ± sd, kg/m^2^	22.64 ± 18.50	21.18 ± 3.95	21.68 ± 17.66	21.68 ± 9.44	0.416
Ln hs-Tnl, mean ± sd	-3.19 ± 1.42	-3.05 ± 1.34	-2.68 ± 1.39	-2.46 ± 1.42	< 0.001
eGFR, mean ± sd, mL/min/1.73 m^2^	103.26 ± 33.38	80.71 ± 26.56	59.19 ± 23.20	32.35 ± 16.38	< 0.001
LnBNP, mean ± sd	6.31 ± 1.30	6.46 ± 1.24	6.65 ± 1.15	6.74 ± 1.23	< 0.001
Charles comorbidity score, mean ± sd	1.64 ± 0.85	1.70 ± 0.86	1.92 ± 0.98	2.19 ± 1.06	< 0.001
LVEF (%), mean ± sd	50.00 ± 13.20	50.94 ± 13.74	49.87 ± 13.31	51.75 ± 12.28	0.596
Creatinine, μmol/L, mean ± sd	63.84 ± 17.32	78.00 ± 22.22	101.89 ± 36.48	191.96 ± 114.04	< 0.001
Sex, n (%)					0.01
Male	177 (36.42%)	210 (43.03%)	211 (42.37%)	232 (46.96%)	
Female	309 (63.58%)	278 (56.97%)	287 (57.63%)	262 (53.04%)	
Age, n (%)					< 0.001
≤ 70 years old	210 (43.21%)	146 (29.92%)	108 (21.69%)	68 (13.77%)	
> 70 years old	276 (56.79%)	342 (70.08%)	390 (78.31%)	426 (86.23%)	
NYHA classification, n (%)					< 0.001
II	97 (19.96%)	94 (19.26%)	86 (17.27%)	66 (13.36%)	
III	277 (57.00%)	255 (52.25%)	241 (48.39%)	242 (48.99%)	
IV	112 (23.05%)	139 (28.48%)	171 (34.34%)	186 (37.65%)	
Vasodilator use, n (%)					0.739
No	167 (34.36%)	166 (34.02%)	169 (33.94%)	155 (31.38%)	
Yes	319 (65.64%)	322 (65.98%)	329 (66.06%)	339 (68.62%)	
Ras-Blocker use, n (%)					< 0.001
No	21 (4.32%)	24 (4.92%)	32 (6.43%)	67 (13.56%)	
Yes	465 (95.68%)	464 (95.08%)	466 (93.57%)	427 (86.44%)	
Inotropes use, n (%)					0.709
No	128 (26.34%)	134 (27.46%)	124 (24.90%)	138 (27.94%)	
Yes	358 (73.66%)	354 (72.54%)	374 (75.10%)	356 (72.06%)	
Diuretic use, n (%)					< 0.001
No	86 (17.77%)	75 (15.40%)	62 (12.53%)	44 (8.91%)	
Yes	398 (82.23%)	412 (84.60%)	433 (87.47%)	450 (91.09%)	
Statin use, n (%)					0.003
No	307 (63.43%)	300 (61.60%)	288 (58.18%)	259 (52.43%)	
Yes	177 (36.57%)	187 (38.40%)	207 (41.82%)	235 (47.57%)	
Number of hospitalizations, n (%)					0.007
≤ 1	437 (89.92%)	462 (94.67%)	471 (94.58%)	451 (91.30%)	
> 1	49 (10.08%)	26 (5.33%)	27 (5.42%)	43 (8.70%)	
28 day all-cause mortality, n (%)					< 0.001
Alive	481 (98.97%)	485 (99.39%)	489 (98.19%)	475 (96.15%)	
Death	5 (1.03%)	3 (0.61%)	9 (1.81%)	19 (3.85%)	
90 day all-cause mortality, n (%)					< 0.001
Alive	481 (98.97%)	485 (99.39%)	486 (97.59%)	473 (95.75%)	
Death	5 (1.03%)	3 (0.61%)	12 (2.41%)	21 (4.25%)	
6 month all-cause mortality, n (%)					0.003
Alive	477 (98.15%)	481 (98.57%)	483 (96.99%)	469 (94.94%)	
Death	9 (1.85%)	7 (1.43%)	15 (3.01%)	25 (5.06%)	

The difference between the sample sum of each column and the total number shown in this table is due to missing data.

BMI, body mass index.

LVEF, left ventricular ejection fraction.

hs-Tnl, High-sensitivity troponin I.

BNP, B-type natriuretic peptide.

NYHA, New York Heart Association.

eGFR, estimated glomerular filtration rate.

We employed a generalized additive model (GAM) to investigate the probability of a non-linear association between CysC and the risk of all-cause death due to the linear character of logistic regression. The inflection point of the curve is calculated using smooth curve fitting and the recursion approach. The relative risk (RR) and 95% confidence interval (CI) on both sides of the inflection point are then calculated using a two-piecewise linear regression model.

We performed the following sensitivity analysis to ensure the study’s robustness: (1) The baseline CysC values were converted to a categorical variable (quartile), and P for trend was calculated to determine whether the CysC RR values were robust; (2) We did not include the missing left ventricular ejection fraction (LVEF) in the full-adjusted model for adjusting due to the large proportion of missing LVEF (missing 1354, 68.88%). However, because LVEF is such an important marker of heart failure, we kept it in the model for sensitivity analysis and tested if the results were still acceptable after it was adjusted. (3) Given that past research has revealed that Cys C is preferable to creatinine. In this study, we employed creatinine as an effect modifier and performed an interaction analysis. Given that the normal value of creatinine in this study was 44-110 μmol/L, we used 110 as the cutoff point and divided it into a normal creatinine group and an elevated creatinine group for the subsequent interaction test. A stratified logistic regression model was used to perform subgroup analysis. An interaction term between creatinine (quartiles) was used to assess for effect modification, followed by a likelihood ratio test. (4) In order to minimize the bias of the noise contained in the data on our findings, we validated the linear and non-linear results by resampling (bootstrap, *n* = 500 times).

All the analyses were performed with the statistical software packages R (^1^The R Foundation) and EmpowerStats (^2^X&Y Solutions, Inc, Boston, MA). P values of less than 0.05 (two-sided) were considered statistically significant.

## Results

### The baseline characteristics of Chinese heart failure patients

Table 1 shows the baseline characteristics of the remaining 1966 cases. 830 (42.22%) of the 1966 patients were men, and 1434 (72.94%) of the patients were 70 or older. The HF death rates were 1.83% (36/1966), 2.09% (41/1966), and 2.85% (56/1966) at 28 days, 90 days, and 6 months, respectively. Patients with higher CysC levels (Q3 and Q4) were older, had higher BNP, creatinine, and hs-Tnl levels, worse renal function, worse cardiac function classification, and higher comorbidity ratings than those with lower CysC levels (Q1 and Q2). Furthermore, the proportion of patients with repeated hospitalization was higher in the Q3 & Q4-CysC groups than in the Q1 & Q2-CysC groups, and the proportion of patients consuming statins, diuretics, and Ras-blockers was substantially higher in the Q3 & Q4-CysC groups than in the Q1 & Q2 CysC groups.

### The association between cystatin C and all-cause mortality obtained from univariate and multivariable logistic regression model

We employed multiple imputation to generate five data sets. However, when we compared these RR values obtained generated from these data sets, there was only a slight difference (data not show). As a result, we ended up solely presenting the results from the completed data.

When CysC was used as a continuous variable in model 1 (Table 2), each increase in CysC of 1 mg/L increased the risk of 28-day mortality by 103% (36 deaths; relative risk[RR], 2.03; 95% confidence interval [CI], 1.62 to 2.54; *P* < 0.001), 90-day mortality by 103% (41 deaths; RR, 2.03; 95%CI, 1.64 to 2.52; *P* < 0.001), and 6-month mortality by 84% (56 deaths; RR, 1.84; 95% CI, 1.51 to 2.23; *P* < 0.001). Furthermore, in model 2, each increase in CysC of 1 mg/L increased the risk of 28-day all-cause mortality (RR, 2.10; 95% CI, 1.68 to 2.63; *P* < 0.001), 90-day mortality (RR, 2.10; 95% CI, 1.68 to 2.63; *P* < 0.001), and 6-month death (RR, 1.86; 95% CI, 1.52 to 2.28; *P* < 0.001). The results of model 2 were about the same as model 1, with both the RR values and the 95% CIs (Table 2). In the fully adjusted model, patients with an increase in CysC had a 42% increase in the risk of 28-day mortality (RR, 1.42; 95%CI, 1.00 to 2.40; *P* = 0.049), an 82% increase in the risk of 90-day mortality (RR, 1.82; 95%CI, 1.13 to 2.95; *P* = 0.014), and a 59% increase in the risk of 6-month mortality (RR, 1.59; 95%CI, 1.06 to 2.38, *P* = 0.024) (Table 2).

**TABLE 2:** The association between cystatin and all-cause mortality using univariate and multivariable binary logistic regression model.

	Model 1 RR, 95%CI, P value	Model 2 RR, 95%CI, P value	Model 3 RR, 95%CI, *P* value
**28 day all-cause mortality**			
Cystatin (continuous variable)	2.03 (1.62, 2.54) < 0.0001	2.05 (1.62, 2.60) < 0.0001	1.42 (1.00, 2.40) 0.049
Cystatin (grouped by quartile)			
Q1 (0.23–1.20)	Reference	Reference	Reference
Q2 (1.21–1.54)	0.60 (0.14, 2.50) 0.4789	0.58 (0.14, 2.45) 0.4568	0.36 (0.06, 2.03) 0.2471
Q3 (1.55–2.19)	1.79 (0.60, 5.38) 0.3001	1.74 (0.57, 5.34) 0.3297	0.49 (0.09, 2.72) 0.4169
Q4 (2.20–7.06)	3.87 (1.43, 10.45) 0.0076	3.71 (1.32, 10.45) 0.0130	0.32 (0.04, 2.38) 0.2673
P for trend	0.0006	0.0011	0.3864
**90 day all-cause mortality**			
Cystatin (continuous variable)	2.03 (1.64, 2.52) < 0.0001	2.10 (1.68, 2.63) < 0.0001	1.82 (1.13, 2.95) 0.0140
Cystatin (grouped by quartile)			
Q1 (0.23–1.20)	Reference	Reference	Reference
Q2 (1.21–1.54)	0.60 (0.14, 2.50) 0.4789	0.60 (0.14, 2.55) 0.4928	0.53 (0.10, 2.75) 0.4512
Q3 (1.55–2.19)	2.40 (0.84, 6.86) 0.1025	2.52 (0.87, 7.33) 0.0902	1.34 (0.30, 6.02) 0.7058
Q4 (2.20–7.06)	4.30 (1.61, 11.49) 0.0037	4.58 (1.65, 12.73) 0.0036	1.04 (0.18, 6.03) 0.9667
P for trend	0.0001	0.0001	0.7452
**6 month all-cause mortality**			
Cystatin (continuous variable)	1.84 (1.51, 2.23) < 0.0001	1.86 (1.52, 2.28) < 0.0001	1.59 (1.06, 2.38) 0.0244
Cystatin (grouped by quartile)			
Q1 (0.23–1.20)	Reference	Reference	Reference
Q2 (1.21–1.54)	0.77 (0.29, 2.09) 0.6095	0.77 (0.28, 2.10) 0.6095	0.59 (0.19, 1.79) 0.3474
Q3 (1.55–2.19)	1.66 (0.72, 3.84) 0.2329	1.68 (0.72, 3.93) 0.2317	0.76 (0.24, 2.38) 0.6402
Q4 (2.20–7.06)	2.84 (1.31, 6.15) 0.0080	2.87 (1.29, 6.41) 0.0101	0.61 (0.16, 2.33) 0.4714
P for trend	0.0010	0.0013	0.6044

RR: relative risk.

CI: Confidence interval.

Model 1: No covariates were adjusted for.

Model 2: We only adjusted for age and sex.

Model 3: We adjusted for all covariates presented in Table 1.

When we transformed CYSC into a categorical variable for sensitivity analysis, we found that the results of the CysC_categorical variable_ were not consistent with the CysC_continuous variable_ (Table 2). In model 3, when compared with the reference (lowest quartile), patients with the highest quartile of CysC showed no significant association with the risk of 28-day, 90-day, and 6-month mortality. This disparity of results (CysC_continuous_ vs. CysC_categorical variables_) suggests that the association of Cysc with death may be non-linear.

### The results of non-linearity of cystatin C and all-cause mortality

**FIGURE 2:**
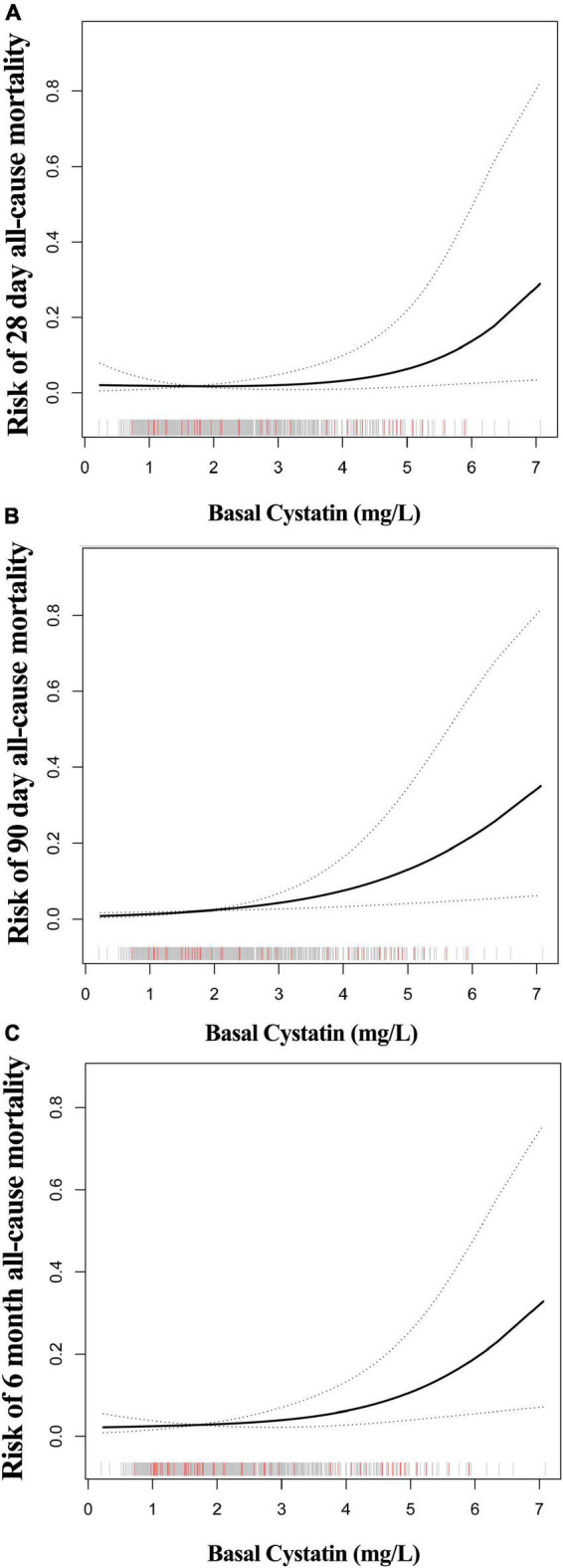
The non-linear relationship between CysC and all-cause mortality of HF. The abscissa represents the CysC. The ordinates of figure **(A–C)** represent the risk of 28-day, 90-day, and 6-month mortality in patients with HF. The middle line represents the trend of all-cause mortality with CysC. The upper and lower lines represent the 95% confidence interval. The indicated lines on the x-axis represent each participant’s CysC level. The red line represents the patient who died. The gray line represents patients who have survived.

A smooth curve and a generalized additive model (GAM) were used to observe the non-linear relationship between CysC and all-cause mortality (Table 3). Our results suggested that the correlations between CysC and the 28-day, 90-day, and 6-month mortality were both non-linear after adjusting for the demographic data, the drug use, the comorbidity score, the organ function status (heart, kidney), and the severity of heart failure (Figure 2). By two-piecewise linear regression model and a recursive algorithm, we calculated the inflection points of CysC to be 2.55 (95%CI: 2.16 to 3.26) for 28-day mortality, 2.58 (95%CI: 2.20 to 3.31) for 90-day mortality, and 2.51 (95%CI: 2.17 to 3.16) for 6-month mortality, respectively. On the left of the inflection points, the RR and 95%CI for CysC and risk of 28-day mortality were 0.47 and 0.13 to 1.66, 0.77 and 0.25 to 2.35 for 90-day mortality, and 0.64 and 0.25 to 1.59 for 6-month mortality, respectively. These results collectively suggest that no significant association between basal CysC levels and HF patient death was seen. On the contrary, the results for the CysC values with patients’ deaths were completely different on the right side of inflection points. We observed that higher CysC was associated with all-cause mortality in HF patients on the right of the inflection point. Our results indicated that each increase in CysC of 1 mg/L increased the risk of 28-day mortality by 107% (RR, 2.07; 95% confidence interval [CI], 1.09 to 3.93; *P* = 0.026), 90-day mortality by 151% (RR, 2.51; 95%CI, 1.38 to 4.57; *P* = 0.003), and 6-month mortality by 125% (RR, 2.25; 95% CI, 1.37 to 3.70; *P* < 0.001).

**TABLE 3:** Exploration of non-linear association between cystatin and all-cause mortality using two-piecewise lienar model.

	28 day all-cause mortality RR, 95%CI, *P* value	90 day all-cause mortality RR, 95%CI, *P* value	6 month all-cause mortality RR, 95%CI, *P* value
Fitting model by standard binary logistic regression model	1.42 (1.00, 2.40) 0.049	1.82 (1.13, 2.95) 0.0140	1.59 (1.06, 2.38) 0.0244
Fitting model by two-piecewise linear model			
Inflection point	2.55 (2.16 to 3.26)	2.58 (2.20 to 3.31)	2.51 (2.17 to 3.16)
≤Inflection point	0.47 (0.13, 1.66) 0.2413	0.77 (0.25, 2.35) 0.6521	0.64 (0.25, 1.59) 0.3308
>Inflection point	2.07 (1.09, 3.93) 0.0266	2.51 (1.38, 4.57) 0.0026	2.25 (1.37, 3.70) 0.0014
P for log likely ratio test	0.05	0.042	0.027

The covariates, which were adjusted for, was the same as model 3.

### The results of sensitivity analyses

(1) On the basis of model 3, we also adjusted LVEF. Despite having bigger effect sizes and broader confidence intervals after controlling for LVEF, CysC’s positive connection with all-cause mortality did not alter, as shown in Supplementary Table 2.
(2) We also looked at whether the non-linear relationship between CysC and all-cause mortality changed after adjusting for LVEF (Supplementary Table 3). The threshold effect still remains without modifying the size of the original inflection point, according to the results of the Two-piecewise linear model (Supplementary Table 3). To the right of the inflection point, we saw greater RR values and wider confidence intervals, which could be attributable to the considerable fall in sample size due to LVEF.
(3) Given that past research has revealed that Cys C is preferable to creatinine, in this study, we employed creatinine as an effect modifier and performed an interaction analysis. Table 4 shows that creatinine, as an effect modifier, had no significant modifying effect on the link of cys-c with death at 90 days or 6 months. To begin with, the interaction P value was not significant (P for interaction = 0.5463 for 90-day mortality and P for interaction = 0.6784 for 6-month mortality). Furthermore, the magnitude of the RR did not alter in trend or direction between the creatinine-normal and creatinine-elevated groups, despite a modest difference. This implies that Cys-C is associated with a higher risk of death in heart failure, regardless of creatinine levels.
(4) We confirmed the adjusted-RR values and confidence intervals using resampling. The data (Supplementary Table 5) reveals that the amplitude of the RR and the 95% confidence interval change only slightly, but the overall trend (direction of the RR) stays constant.

**TABLE 4:** The interaction analyses using creatinine as effect modifier.

Model	X65.CS: <110	X65.CS: =110	P interaction
30-day mortality	0.71 (0.15, 3.38) 0.6670	1.92 (1.27, 2.92) 0.0021	0.1959
90-day mortality	2.75 (1.13, 6.66) 0.0254	2.03 (1.36, 3.02) 0.0005	0.5463
6-month mortality	2.25 (1.05, 4.84) 0.0377	1.88 (1.32, 2.67) 0.0005	0.6784

Adjusted covariates as in model 3 except for creatinine.

## Discussion

In a large retrospective cohort of Chinese HF patients, we investigated the relationship between baseline CysC and death. The findings revealed that the link between CysC and all-cause mortality had a threshold effect, meaning that the link between CysC and mortality risk was only found when CysC was larger than 2.5 mg/L. Every 1 mg/L rise in CysC was associated with a doubling of the risk of all-cause death when CysC was between 2.52 and 7.06 mg/L.

The relationship between CysC and heart failure has been extensively researched. Data from earlier studies appear to indicate that CysC is positively associated with the severity of heart failure and the risk of poor prognosis (18–21). A meta-analysis of ten studies (sample sizes ranging from 195 to 628 cases) on basal CysC levels in HF patients discovered that higher CysC was associated with higher of all-cause death and rehospitalization (14). However, only one of the ten investigations in this meta-analysis was conducted on a Chinese population (22). Furthermore, non-linearity was not taken into account in any of these studies. ZQ Yao et al. (23) reported in a case-control study published in 2021 that patients with major adverse cardiovascular events (MACE) had considerably higher levels of Cys-C than those in the research group who did not have MACE. In contrast, the conclusions of this study were based on a univariate analysis with no adjustment for potential confounders. Our findings are consistent with previous research. According to the findings of multivariate logistic regression, high CysC was always associated with an increased risk of all-cause death in our study. We, on the other hand, go beyond the past investigations. In our investigation, we did not limit ourselves to linear linkages; instead, we looked into non-linear possibilities. To our knowledge, just one previous non-linear CysC association with poor HF outcomes has been reported (24). The study, however, only comprised 418 patients, and the identified non-linear connections were not explained. Our study not only discovered non-linear relationships, but it also identified the inflection point and quantified the threshold impact. This will provide researchers with a fresh look at the link between CysC and heart failure in clinical practice. Furthermore, it contributes to the formulation of more personalized and appropriate healthcare decisions.

Some of the advantages of our research include: (1) The current study has a large sample size, allowing us to analyze the relationship between CysC and HF mortality; (2) we used advanced algorithms (GAM model and two-piecewise linear model) to detect the true association between CysC and the risk of all-cause death in Chinese HF patients. (3) A number of sensitivity studies supported our findings. (4) We validated prior findings that cys-C outperforms creatinine in predicting the chance of death from heart failure using interaction testing. Our research, however, has certain drawbacks. To begin, all of our research participants were Chinese. This complicates applying our findings to other races. Second, we couldn’t account for confounders that couldn’t be measured. Third, the LVEF is an important covariate. However, this variable had a large missing ratio in the original database. If we include it in the model, we will lose a lot of sample sizes. This will undoubtedly affect the correctness of our assessments. As a result, we conducted a sensitivity analysis. Even after adjusting for LVEF, the overall trend of the data did not change, according to the results of the sensitivity analysis (Supplementary Tables 2, 3). (4)For analyzing survival data, proportional hazard models outperform logistic regression algorithmically. However, because the original data only included 28-day, 90-day, and 6-month survival status, but no comparable follow-up times or total survival status, we were unable to build a proportional hazards model. (5) Although the data builder uploaded 28-day, 90-day, and 180-day death states for the entire database, they did not specify whether left-censored data (follow-up stopped before the scheduled follow-up time point and the patient was still alive) were excluded. In this study, however, the correlation of CysC with all three outcome variables was consistent and robust. Furthermore, even if there was left-censored data that biased the results, the bias was in the opposite direction of our RR values, resulting in stronger results. (6) We controlled for the number of readmissions but not the reason for the readmission (cardiovascular vs. non-cardiovascular). This is due to the fact that this indicator was not included in the original database. However, sensitivity analysis found little difference in RR with or without readmission adjustment (Supplementary Table 4).

## Conclusion

According to our findings, cystatin levels of 2.5 mg/l may be a dangerous threshold for Chinese HF patients. Because the risk of all-cause death increases by more than one-fold for every 1 mg/L increase in CysC above this threshold.

## Data availability statement

The original contributions presented in this study are included in the article/Supplementary material, further inquiries can be directed to the corresponding author.

## Ethics statement

The studies involving human participants were reviewed and approved by Zigong Fourth People’s Hospital (Approval Number: 2020-010). The ethics committee waived the requirement of written informed consent for participation.

## Author contributions

JC contributed to the research design and data analysis. TZ contributed to the writing of the manuscript. Y-LH participated in manuscript writing and data analysis. A-MT contributed to the review of the manuscript and the interpretation of clinical significance. All authors contributed to the article and approved the submitted version.

## Footnotes

1
http://www.R-project.org

2
http://www.empowerstats.com

## Supplementary material

Supplementary Figure 1 It shows the frequency distribution (histogram) of Cys-C in the surviving and non-surviving groups at each time point, and figures **(A–F)** show the frequency distribution of Cys-C in patients with different survival status at each time point.

## References

[1] McEwan P Darlington O McMurray JJV Jhund, Docherty KF Böhm M Cost-effectiveness of dapagliflozin as a treatment for heart failure with reduced ejection fraction: a multinational health-economic analysis of DAPA-HF. *Eur J Heart Fail.* (2020) 22:2147–56. 10.1002/ejhf.1978 32749733 PMC7756637

[2] Brettell R Soljak M Cecil E Cowie MR Tuppin P Majeed A. Reducing heart failure admission rates in England 2004-2011 are not related to changes in primary care quality: national observational study. *Eur J Heart Fail.* (2013) 15:1335–42. 10.1093/eurjhf/hft107 23845798 PMC3834843

[3] Hao G Wang X Chen Z Zhang L Zhang Y Wei B Prevalence of heart failure and left ventricular dysfunction in China: the China Hypertension Survey, 2012-2015. *Eur J Heart Fail.* (2019) 21:1329–37. 10.1002/ejhf.1629 31746111

[4] Cook C Cole G Asaria P Jabbour R Francis DP. The annual global economic burden of heart failure. *Int J Cardiol.* (2014) 171:368–76. 10.1016/j.ijcard.2013.12.028 24398230

[5] van Dijk B Lemans J Hoogendoorn RM Dadachova E de Klerk JM Vogely HC Treating infections with ionizing radiation: a historical perspective and emerging techniques. *Antimicrob Resist Infect Control.* (2020) 9:121. 10.1186/s13756-020-00775-w 32736656 PMC7393726

[6] Heart Failure Group of Chinese Society of Cardiology of Chinese Medical Association, Chinese Heart Failure Association of Chinese Medical Doctor Association, Editorial Board of Chinese Journal of Cardiology. Chinese guidelines for the diagnosis and treatment of heart failure 2018. *Zhonghua Xin Xue Guan Bing Za Zhi.* (2018) 46:760–89. 30369168 10.3760/cma.j.issn.0253-3758.2018.10.004

[7] Pérez Calvo JI Josa Laorden C Giménez López I. Renal function assessment in heart failure. *Rev Clin Esp.* (2017) 217:267–88. 10.1016/j.rceng.2017.03.003 28258719

[8] Smith GL Lichtman JH Bracken MB Shlipak MG Phillips CO DiCapua P Renal impairment and outcomes in heart failure: systematic review and meta-analysis. *J Am Coll Cardiol.* (2006) 47:1987–96. 10.1016/j.jacc.2005.11.084 16697315

[9] Hsu CY Chertow GM Curhan GC. Methodological issues in studying the epidemiology of mild to moderate chronic renal insufficiency. *Kidney Int.* (2002) 61:1567–76. 10.1046/j.1523-1755.2002.00299.x 11967006

[10] Fan L Inker LA Rossert J Froissart M Rossing P Mauer M Glomerular filtration rate estimation using cystatin C alone or combined with creatinine as a confirmatory test. *Nephrol Dial Transplant.* (2014) 29:1195–203. 10.1093/ndt/gft509 24449101 PMC4471437

[11] Shlipak MG Katz R Fried LF Jenny NS Stehman-Breen C Newman AB Cystatin-C and mortality in elderly persons with heart failure. *J Am Coll Cardiol.* (2005) 45:268–71. 10.1016/j.jacc.2004.09.061 15653026

[12] Zhang Z Lu B Sheng X Jin N. Cystatin C in prediction of acute kidney injury: a systemic review and meta-analysis. *Am J Kidney Dis.* (2011) 58:356–65. 10.1053/j.ajkd.2011.02.389 21601330

[13] Madero M Sarnak MJ. Association of cystatin C with adverse outcomes. *Curr Opin Nephrol Hypertens.* (2009) 18:258–63. 10.1097/MNH.0b013e328326f3dd 19374014 PMC2890263

[14] Chen S Tang Y Zhou X. Cystatin C for predicting all-cause mortality and rehospitalization in patients with heart failure: a meta-analysis. *Biosci Rep.* (2019) 39:BSR20181761. 10.1042/BSR20181761 30643006 PMC6361773

[15] Collins GS Reitsma JB Altman DG Moons KG. Transparent reporting of a multivariable prediction model for individual prognosis or diagnosis (TRIPOD): the TRIPOD statement. *BMJ.* (2015) 350:g7594. 10.1136/bmj.g7594 25569120

[16] Zhang Z Cao L Chen R Zhao Y Lv L Xu Z Electronic healthcare records and external outcome data for hospitalized patients with heart failure. *Sci Data.* (2021) 8:46. 10.1038/s41597-021-00835-9 33547290 PMC7865067

[17] Ponikowski P Voors AA Anker SD Bueno H Cleland JG Coats AJ 2016 ESC Guidelines for the diagnosis and treatment of acute and chronic heart failure: the Task Force for the diagnosis and treatment of acute and chronic heart failure of the European Society of Cardiology (ESC). Developed with the special contribution of the Heart Failure Association (HFA) of the ESC. *Eur J Heart Fail.* (2016) 18:891–975. 10.1002/ejhf.592 27207191

[18] Breidthardt T Sabti Z Ziller R Rassouli F Twerenbold R Kozhuharov N Diagnostic and prognostic value of cystatin C in acute heart failure. *Clin Biochem.* (2017) 50:1007–13. 10.1016/j.clinbiochem.2017.07.016 28756070

[19] Xu CC Fu GX Liu QQ Zhong Y. Association between cystatin C and heart failure with preserved ejection fraction in elderly Chinese patients. *Z Gerontol Geriatr.* (2018) 51:92–7. 10.1007/s00391-016-1058-5 27206415

[20] Huerta A López B Ravassa S San José G Querejeta R Beloqui Ó Association of cystatin C with heart failure with preserved ejection fraction in elderly hypertensive patients: potential role of altered collagen metabolism. *J Hypertens.* (2016) 34:130–8. 10.1097/HJH.0000000000000757 26575701

[21] Rafouli-Stergiou P Parissis J Farmakis D Bistola V Nikolaou M Vasiliadis K Prognostic value of in-hospital change in cystatin C in patients with acutely decompensated heart failure and renal dysfunction. *Int J Cardiol.* (2015) 182:74–6. 10.1016/j.ijcard.2014.12.135 25576725

[22] Ruan ZB Zhu L Yin YG Chen GC. Cystatin C. N-terminal probrain natriuretic peptides and outcomes in acute heart failure with acute kidney injury in a 12-month follow-up: insights into the cardiorenal syndrome. *J Res Med Sci.* (2014) 19:404–9. 25097621 PMC4116570

[23] Yao Z Li G Li G. Correlation between serum urea nitrogen, cystatin C, homocysteine, and chronic heart failure. *Am J Transl Res.* (2021) 13:3254–61. 34017496 PMC8129353

[24] Wu X Xu G Zhang S. Association between cystatin c and cardiac function and long-term prognosis in patients with chronic heart failure. *Med Sci Monit.* (2020) 26:e919422. 10.12659/MSM.919422 32062670 PMC7043349

