# Supplementary figures and images for: Non-linear association of cystatin C and all-cause mortality of heart failure: A secondary analysis based on a published database
Source: Front Cardiovasc Med. 2022 Sep 6;9:930498. doi: 10.3389/fcvm.2022.930498 (PMC9488665; doi:10.3389/fcvm.2022.930498)

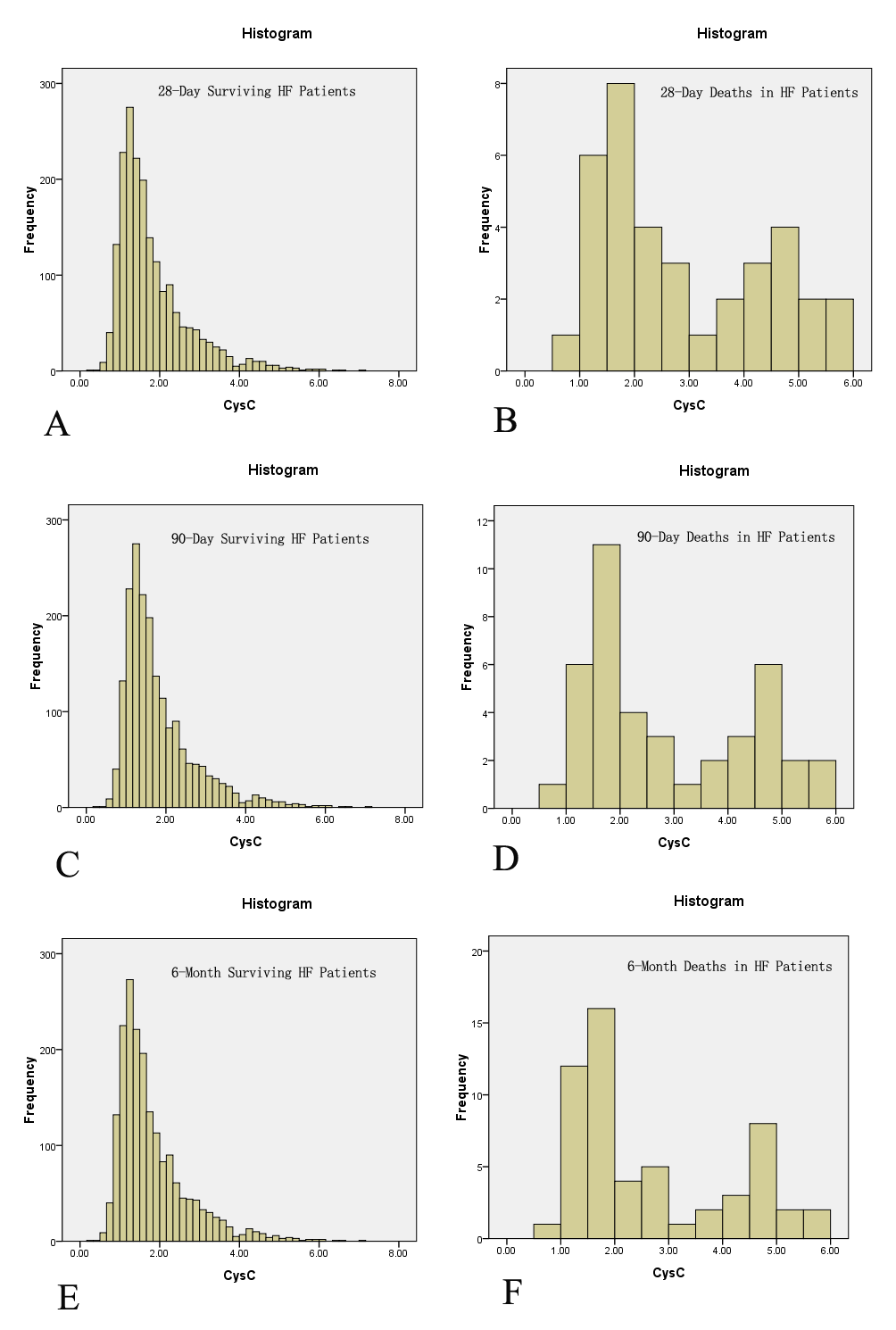

Supplement: Supplementary Figure 1 — It shows the frequency distribution (histogram) of Cys-C in the surviving and non-surviving groups at each time point, and figures (A–F) show the frequency distribution of Cys-C in patients with different survival status at each time point. [file Image_1.TIF]
