# Supplementary material for: Non-linear association of cystatin C and all-cause mortality of heart failure: A secondary analysis based on a published database
Source: Front Cardiovasc Med. 2022 Sep 6;9:930498. doi: 10.3389/fcvm.2022.930498 (PMC9488665; doi:10.3389/fcvm.2022.930498)
Supplement: Supplementary file 2 [file Table_1.DOCX]

Supplemental table 1: The description of missing data

| Variables | Without missing | Missing | missing rate (%) |
| --- | --- | --- | --- |
| Sex | 1966 | 0 | 0 |
| Age | 1966 | 0 | 0 |
| BMI | 1966 | 0 | 0 |
| NYHA classification | 1966 | 0 | 0 |
| Vasodilator use | 1966 | 0 | 0 |
| Ras-blocker use | 1966 | 0 | 0 |
| Inotropes use | 1966 | 0 | 0 |
| Diuretic use | 1960 | 6 | 0.31 |
| Ln hs-Tnl | 1899 | 67 | 3.41 |
| Number of hospitalizations | 1995 | 0 | 0 |
| eGFR | 1927 | 39 | 1.98 |
| BNP | 1940 | 26 | 1.32 |
| 28 day all-cause mortality | 1995 | 0 | 0 |
| 90 day all-cause mortality | 1995 | 0 | 0 |
| 6 month all-cause mortality | 1995 | 0 | 0 |
| Stain use | 1960 | 6 | 0.31 |
| Charlson Comorbidity index score | 1961 | 5 | 0.25 |

Supplemental table 2: The association between Cystatin and mortality after additionally adjusting for LVEF

|  | Fully-adjusted model  OR, 95%CI, P value | Fully-adjusted+LVEF  OR, 95%CI, P value |
| --- | --- | --- |
| 28 day all-cause mortality |  |  |
| Cystatin (continuous variable) | 1.62 (1.04, 2.53) 0.0335 | 6.17 (1.78, 21.36) 0.0041 |
| Cystatin (grouped by quartile) |  |  |
| Q1 (0.23-1.20) | Reference | Reference |
| Q2 (1.21-1.54) | 0.29 (0.05, 1.66) 0.1660 | 0.39 (0.01, 10.08) 0.5689 |
| Q3 (1.55-2.19) | 0.33 (0.06, 1.73) 0.1895 | 0.14 (0.00, 6.14) 0.3070 |
| Q4 (2.20-7.06) | 0.23 (0.03, 1.69) 0.1502 | 0.14 (0.00, 13.32) 0.3988 |
| P for trend | 0.2626 | 0.55 (0.13, 2.35) 0.4159 |
| 90 day all-cause mortality |  |  |
| Cystatin (continuous variable) | 1.86 (1.24, 2.77) 0.0025 | 6.17 (1.78, 21.36) 0.0041 |
| Cystatin (grouped by quartile) |  |  |
| Q1 (0.23-1.20) | Reference | Reference |
| Q2 (1.21-1.54) | 0.47 (0.09, 2.47) 0.3753 | 0.39 (0.01, 10.08) 0.5689 |
| Q3 (1.55-2.19) | 0.99 (0.23, 4.36) 0.9908 | 0.14 (0.00, 6.14) 0.3070 |
| Q4 (2.20-7.06) | 0.85 (0.15, 5.01) 0.8614 | 0.14 (0.00, 13.32) 0.3988 |
| P for trend | 0.8727 | 0.4159 |
| 6 month all-cause mortality |  |  |
| Cystatin (continuous variable) | 1.60 (1.15, 2.24) 0.0058 | 6.17 (1.78, 21.36) 0.0041 |
| Cystatin (grouped by quartile) |  |  |
| Q1 (0.23-1.20) | Reference | Reference |
| Q2 (1.21-1.54) | 0.55 (0.18, 1.67) 0.2901 | 0.39 (0.01, 10.08) 0.5689 |
| Q3 (1.55-2.19) | 0.65 (0.21, 1.99) 0.4511 | 0.14 (0.00, 6.14) 0.3070 |
| Q4 (2.20-7.06) | 0.57 (0.15, 2.20) 0.4177 | 0.14 (0.00, 13.32) 0.3988 |
| P for trend | 0.5523 | 0.4159 |

Abbreviation:

OR: odds ratio

CI: Confidence interval

Fully-adjusted model: We adjusted for all covariates presented in table 1.

Supplemental Table3: Exploration of nonlinear association between Cystatin and all-cause mortality using two-piecewise lienar model (additionally adjusting for LVEF)

|  | 28 day all-cause mortality  OR, 95%CI, P value | 90 day all-cause mortality  OR, 95%CI, P value | 6 month all-cause mortality  OR, 95%CI, P value |
| --- | --- | --- | --- |
| Fitting model by standard binary logistic regression model | 6.17 (1.78, 21.36) 0.0041 | 6.17 (1.78, 21.36) 0.0041 | 6.17 (1.78, 21.36) 0.0041 |
| Fitting model by two-piecewise linear model |  |  |  |
| Inflection point | 2.55 | 2.58 | 2.53 |
| ≤ inflection point | 0.59 (0.04, 8.31) 0.6935 | 0.60 (0.04, 8.34) 0.7042 | 0.58 (0.04, 8.31) 0.6874 |
| > inflection point | 18.65 (2.67, 130.09) 0.0032 | 19.48 (2.71, 139.99) 0.0032 | 18.13 (2.65, 123.93) 0.0031 |
| P for log likely ratio test | 0.040 | 0.038 | 0.041 |

The covariates, which were adjusted for, was the same as fully-adjusted model

Supplemental table 4: Change in RR with/without adjustment for number of stays

|  | Model 3  OR, 95%CI, P value | Model 3 not adjusted for hospital readmission  OR, 95%CI, P value |
| --- | --- | --- |
| 28 day all-cause mortality |  |  |
| Cystatin (continuous variable) | 1.62 (1.04, 2.53) 0.0335 | 1.63 (1.05, 2.54) 0.0310 |
| 90 day all-cause mortality |  |  |
| Cystatin (continuous variable) | 1.86 (1.24, 2.77) 0.0025 | 1.84 (1.23, 2.74) 0.0028 |
| 6 month all-cause mortality |  |  |
| Cystatin (continuous variable) | 1.60 (1.15, 2.24) 0.0058 | 1.60 (1.15, 2.24) 0.0057 |

Supplemental table 5: Adjusted-RR values before and after resampling verification

|  | Pre-validation Model 3  RR, 95%CI, P value | Post-validation Model 3*  RR, 95%CI, P value |
| --- | --- | --- |
| 28 day all-cause mortality |  |  |
| Cystatin (continuous variable) | 1.42 (1.00, 2.40) 0.049 | 1.42 (0.91, 2.94) 0.265 |
| 90 day all-cause mortality |  |  |
| Cystatin (continuous variable) | 1.82 (1.13, 2.95) 0.0140 | 1.74 (1.08, 3.32) 0.0112 |
| 6 month all-cause mortality |  |  |
| Cystatin (continuous variable) | 1.59 (1.06, 2.38) 0.0244 | 1.55 (0.98, 2.53) 0.062 |

"*" indicates that the model was subjected to resampling (bootstrap, times=500) for validation.

Model 3 adjusted for all covariates presented in Table 1
